# The role of Xpert MTB/RIF in diagnosing pulmonary tuberculosis in post-mortem tissues

**DOI:** 10.1038/srep20703

**Published:** 2016-02-10

**Authors:** Alberto L. García-Basteiro, Mamudo R. Ismail, Carla Carrilho, Esperança Ussene, Paola Castillo, Dércio Chitsungo, Cristina Rodríguez, Lucília Lovane, Andrea Vergara, Elisa López-Varela, Inacio Mandomando, Cesaltina Lorenzoni, Jaume Ordi, Clara Menéndez, Quique Bassat, Miguel J. Martínez

**Affiliations:** 1 Centro de Investigação em Saúde de Manhiça (CISM), Maputo, Mozambique; 2 ISGlobal, Barcelona Ctr. Int. Health Res. (CRESIB), Hospital Clínic - Universitat de Barcelona, Barcelona, Spain; 3 Amsterdam Institute for Global Health and Development (AIGHD), Amsterdam, The Netherlands; 4 Department of Pathology, Faculty of Medicine/Eduardo Mondlane University and Maputo Central Hospital, Maputo, Mozambique; 5 Department of Pathology, Hospital Clinic, Universitat de Barcelona, Spain; 6 Department of Clinical Microbiology, Hospital Clinic, Universitat de Barcelona, Spain; 7 Instituto Nacional de Saúde (INS), Ministério da Saúde, Maputo, Moçambique

## Abstract

The extent to which the Xpert MTB/RIF (Gene Xpert) contributes to tuberculosis (TB) diagnosis in samples other than sputum and cerebrospinal fluid remains uncertain. We aimed to assess the role of Xpert MTB/RIF for detecting *M. tuberculosis* in post-mortem tissues. We conducted a study among 30 complete diagnostic autopsies (CDA) performed at the Maputo Central Hospital (Mozambique). Lung tissues were screened for TB in all cases. In addition other tissues were tested when compatible lesions were identified in the histological exam. We used in-house real time PCR and LAMP assays to confirm the presence of *M. tuberculosis* DNA. The diagnosis of tuberculosis at death was established based on microbiological and histopathological results. Eight out of 30 cases (26.7%) were diagnosed of tuberculosis. Xpert had a sensitivity to detect TB in lung tissue of 87.5% (95% CI 47.3–99.7) and a specificity of 95.7% (95% CI: 78.1–99.9). In-house DNA amplification methods and Xpert showed 93.6% concordance for lung tissue and 100% concordance for brain and liver tissues. The final cause of death was attributable to tuberculosis in four cases. Xpert MTB/RIF may represent a valuable, easy-to perform technique for post-mortem TB diagnosis.

Tuberculosis (TB) remains a leading cause of morbidity and mortality, especially in low and middle-income countries. The World Health Organization (WHO) estimates that around one third of all TB cases remain undiagnosed or unreported^1^. Having highly sensitive and specific TB diagnostic tests would contribute to achieve the 90% reduction in TB incidence by 2035, as established by the End-TB strategy^2^. Fortunately, after many years of limited progress on TB diagnostics, the last decade has witnessed renewed efforts on TB diagnostics development and assay evaluation^3^.

In 2010, the Xpert MTB/RIF assay (Cepheid, Sunnyvale, CA, USA. hereafter referred to as Xpert), a rapid, real time PCR-based diagnostic platform, was endorsed for the diagnosis of TB in sputum by the WHO^4^. A recent systematic review showed a pooled sensitivity of 89% and specificity of 99% for pulmonary TB diagnosis, against culture as gold standard^5^. The high sensitivity for TB diagnosis (especially for HIV infected TB cases) and the ability to detect mutations to *rpoB* gene (which confers resistance to Rifampicin, proxy of multi drug resistance) are key features that led to the roll out of this technology throughout the world. Several studies have analyzed the potential role of Xpert in blood^6^, cerebrospinal fluid^7^, urine^8^ or stool^9^, and other specimens^10^, which are potential alternatives for the diagnosis of TB, especially in the case of extra-pulmonary TB, HIV-TB co-infected patients or childhood TB. Although the latest WHO guidelines recommend that the Xpert assay should be used for testing cerebrospinal fluid specimens from patients presumed to have TB meningitis^11^, the extent to which Xpert contributes to TB diagnosis in other samples remains uncertain. Likewise, the role of Xpert for TB diagnosis in postmortem tissues has been underexplored.

Mozambique is one of the 22 TB high burden countries facing many challenges to achieve successful control^1^,^12^, showing high incidence rates, especially among HIV-infected patients^13^,^14^. The case detection rate has been estimated at 37%, thus very low certainty exists on mortality estimates^1^. In Mozambique as well as other high TB burden countries, a rapid, easy to perform and reliable technique that helps to establish TB diagnosis at death, could have a significant impact on the accuracy of global TB mortality statistics, and thus on health planning. Therefore, the objective of this study was to explore the utility of the Xpert assay for TB diagnosis in postmortem tissues in a high TB/HIV prevalence area.

## Methods

This study is part of a large project aimed at validating minimally invasive autopsy (MIA) procedures for post-mortem investigation in Mozambique and Brazil^15^. The study included 30 adult patients who died at the Maputo Central Hospital (MCH) in November 2013. Briefly, assessment of the cause of death was performed to the two most recent deaths each morning (less than 24 hours from time of death) occurring in patients admitted for any cause at the MCH (excluding accidents or traumatic deaths), whose family had provided oral informed consent. A complete diagnostic autopsy (CDA) was performed to all cases. Further details on patient recruitment and methods of the larger study can be found elsewhere^16^.

### Sample collection

Samples from lung, brain and liver were routinely collected from all cases during CDA. All samples from lung tissue were analyzed for molecular testing. In addition, any macroscopic lesion suggestive of tuberculosis in any of these organs was sampled and analysed with molecular tests. A first macroscopic evaluation of the whole organ was conducted and then multiple sections of the organ were performed in order to sample lesions or areas with suspected lesions.

### Sample Processing

For molecular microbiological analyses lung, brain and liver tissue samples were collected in tubes containing lysis buffer (ATL buffer, Qiagen, Hildren, Germany). The volume of the tissue samples corresponded approximately to 0,5 ml. Tissue samples were thawed then homogenized using a handheld rotor-stator homogenizer (Tissue Ruptor, Qiagen), and disposable probes for each tissue sample processed. Nucleic acid extractions (DNA + RNA) were performed using a semi-automated system (Qiacube, Qiagen). In house real-time PCR was used following the procedures described by Espasa *et al*^17^. Loop-mediated isothermal amplification (LAMP) assay^18^ was used to confirm all positive conventional real time PCR results following the procedures described by Iwamoto and colleagues^18^. For the Xpert assay, sample preparation buffer was added to the homogenized samples in a 4:1 ratio, incubated at room temperature, and subsequently processed for Xpert testing as described by manufacturers^19^.

### Tuberculosis case definitions

Tuberculosis disease at death was defined as presence of TB DNA through both in-house molecular methods (real time PCR and LAMP) in a given sample in addition to histologically compatible lesions (including inflammatory response). This case definition was considered the gold standard for the assessment of Xpert performance.

Tuberculosis as cause of death was defined when TB disease was present at death and the review of the entire post-mortem results and clinical data deemed this pathology as the most likely cause.

### Data collection and analysis

We collected clinical information from all subjects using a standardized form, including age, sex, HIV status, previous TB history, current or past anti-tuberculosis treatment, TB compatible symptoms, among others. HIV status was confirmed postmortem by an automated method (ADVIA Centaur HIV 1/0/2 Enhanced assay, Siemens Healthcare Diagnostics) and by PCR using the Cobas TaqMan HIV-1 test v2.0 (Roche Molecular Systems). The Xpert assay reports the detection of DNA from *Mycobacterium* tuberculosis complex (MTBC), the PCR cycle threshold (C_T_) as well as the presence of mutations of the rpoB gene. The results obtained after histopathological and microbiological examination were discussed by the study team composed by a pathologist, microbiologist, and epidemiologist and a physician specialized in tropical medicine. This team was blinded to the Xpert assay results, and based on the histological and microbiological results, determined the final cause of death for each case. The lab technologists who performed the Xpert tests were different from those participating in the overall study, and were not aware of the actual diagnosis of the cases. All clinical data and the results from the histopathological and microbiological examination of the samples were entered into a Microsoft Excel designed spreadsheet and then analyzed using Stata 13 (Stata Corp., College Station, TX). The diagnostic accuracy of the Xpert assay on lung tissue against the composite reference standard for pulmonary TB disease (the case definition) was calculated. The point sensitivity and specificity of Xpert was given with 95% exact binomial confidence intervals.

### Ethical considerations

The protocol of the main study was approved by the National Mozambican Ethics Committee (ref. 342/CNBS/13) and the Ethics Committee of the Hospital Clinic of Barcelona, Spain. All study procedures and methods were carried out in accordance with the approved protocols and Ethics Committees’ existing guidelines.

## Results

Out of the 30 cases, 12 (40%) were female and 19 (63.3%) were HIV-infected (higher HIV prevalence for males than for females: 77.8% vs 41.7% respectively, p = 0.04). The mean age at death was 40.3 years (range 17–76).

Eight of these 30 patients were diagnosed of TB (26.7%) according to the study case definition. Six (75.0%) of them were female and 6 (75.0%) were HIV positive. Clinical pre-mortem TB diagnosis was made in only one case, which was also the only case with documented anti-tuberculosis therapy. TB was the cause of death in 4 out of the 8 cases (13.3% of whole series). Among the 19 HIV infected cases, 3 (15.8%) had TB as cause of death Table 1 shows TB disease findings among the study cases.

Seven of the eight cases with TB at death tested positive with Xpert in lung tissue. There was an Xpert false negative case (case 10) in an HIV-infected patient whose cause of death was septic bacterial pneumonia due to *Acinetobacter baumanni*, but with both real time PCR and LAMP assays positive for TB. There was one case, which tested positive with Xpert and LAMP in lung tissue, but negative with in house PCR (all tests positive in brain tissue). No false positive results for Xpert were recorded. Thus, concordance between the results of Xpert and real time PCR occurred in 28 out of the 30 lung tissue cases (93.6%). The sensitivity of the Xpert assay to detect lungTB disease was 87.5% (95% CI 47.3–99.7) and the specificity was of 95.6% (95.7% CI: 78.1–99.9). Both in-house molecular methods and Xpert showed 100% concordance for liver (1 positive sample out of 1 tested) and brain tissues (3 positive for both methods out of 7 tested). None of the cases tested through Xpert showed mutation in the rpoB gene. Table 2 shows the results for Xpert and in-house molecular methods for all tuberculosis cases.

## Discussion

To our knowledge this is the first study comparing the diagnostic accuracy of Xpert MTB/RIF with conventional real time PCR for TB diagnosis in tissue samples from autopsy cases of unknown cause of death. The study shows that 7 out of 8 TB cases were identified with Xpert, thus this assay may represent a valuable, easy-to perform technique for post-mortem TB diagnosis. Moreover, this study showed a high hidden burden of TB disease among cases with unknown cause of death, which draws attention as to the insufficient accuracy of the current methods used to estimate TB mortality worldwide.

The results of this study, which add to the limited knowledge of Xpert performance in non-liquid extrapulmonary samples, show that Xpert may be a valuable assay in detecting MTB DNA from post-mortem lung, brain or liver tissue. These findings also support those of a recent autopsy study showing that all TB cases diagnosed through histopathological examination were also Xpert positive in postmortem tissues, although that study did not use a conventional method to confirm TB^20^. However, another study by Yagmur and colleagues comparing real time PCR vs Xpert in paraffin-embedded tissues showed limited sensitivity of both tests (against TB defined by histopathologic findings), which could be attributable to the method of preserving the samples^21^. Although of small sample size, some reports have shown the good performance of Xpert in non-liquid specimens, such as lymphadenopathies^22^, joint and bone^23^, or other tissues^24^,^25^. According to the C_T_ values, Xpert detected *M. tuberculosis* in most of the samples with high, low and medium detection levels, potentially reflecting a high bacillary load in the tissues analysed^26^. One case with high C_T_ value in lung tissue, which arguable could have been considered a false positive, had a low C_T_ in brain tissue and also showed acid fast bacilli and TB histopathological compatible lesions.

In this study there were four cases of TB disease without any of the clinical diagnoses being TB and with a cause of death other than TB. The latter could be explained by the unspecific presentation of TB, often atypical in HIV-infected patients (all of them were HIV-infected), or perhaps masked by symptoms caused by the actual cause of death. In one of these cases the real time PCR and LAMP were positive in lung tissue without any typical TB compatible lesions, but with inflammatory response at histological examination. Histologically compatible lesions might not have been found due to the strong immunosuppression associated to advanced AIDS, although not all lung tissue was examined under the microscope due to the absence of macroscopically compatible lesions. Although some might argue that this could be a case of latent TB infection^27^, we believe we cannot diagnose it with currently available diagnostic tools^28^. With a bacterial load high enough to be detected by real time PCR and LAMP, we suggest this could represent a case of emerging TB disease, one of the middle states of the continuum whose ends are latent TB infection and active TB disease^29^. Regardless of whether any of these four cases could have been diagnosed of TB before death, these results show a considerable hidden burden of TB disease at death. Moreover, only one of the 4 cases having died of TB had been diagnosed with TB pre-mortem. This goes in line with findings from a recent autopsy study in Zambian adults showing that 26% of cases with TB had not been clinically diagnosed^20^. Importantly, three non-TB cases were misdiagnosed as tuberculosis by clinicians (2 as main diagnosis and 1 as main secondary diagnosis), but no evidence of TB was found in the post-mortem analysis.

This study had several limitations. First, the sample size is limited and hinders any robust conclusions, especially for tissues other than lungs. Second, liquid culture was not used in this study, which would have been a better gold standard against to compare both Xpert and in-house molecular methods. Likewise, the use of genome sequencing would also have allowed to better characterize the MTB strains involved, as well as provide further robustness to the gold standard. However this was not possible due to the agreed conditions for shipment and analysis of samples of the larger autopsy study. Third, the fact that Xpert is a real time PCR, and the principals of molecular detection are somewhat similar to the conventional PCR, could have led to certain incorporation bias. We believe that the composite nature of our gold standard could have contribute to minimize the potential bias of our Xpert performance estimates. Lastly, it was difficult to infer whether the sample processing strategy was suboptimal (ie. ratio of inactivation buffer/sample, sample volume) or to what extend the conditions of sample storage (in lysis buffer solution) could affect the performance of both the tests. Taking into account the histopathological examination and the excellent concordance of both tests, it is unlikely that the latter limitation would significantly affect the study results.

In conclusion, Xpert seems to be a reliable method for TB post-mortem diagnosis, more rapid and easy to perform than conventional molecular methods. This study also shows that in high TB and HIV endemic countries, TB disease may be significantly underdiagnosed and often misdiagnosed. For individual and public health benefits, there is a need to develop and more widely apply new methods to reliably estimate TB mortality.

## Additional Information

**How to cite this article**: García-Basteiro, A. L. *et al.* The role of Xpert MTB/RIF in diagnosing pulmonary tuberculosis in post-mortem tissues. *Sci. Rep.* **6**, 20703; doi: 10.1038/srep20703 (2016).

## Figures and Tables

**Table 1:** Characteristics and cause of death of patients included in the study.

Case #	Age	Sex	HIV status	Cause of death	TB Disease at death	Positive Organs*	Histology	Any clinical Diagnosis of TB
**1**	**27**	**Male**	**Infected**	**Rhizopus oryzae cerebral mucormycosis**	**Present**	**Lung**	**Pulmonary necrotizing granulomas**	**No**
2	76	Male	Uninfected	Cerebral infarction				
3	30	Male	Uninfected	Hepatocellular carcinoma				
4	19	Female	Uninfected	Cerebral toxoplasmosis				
5	24	Female	Uninfected	Rheumatic valvular heart disease				
6	28	Female	Infected	Disseminated Kaposi’s sarcoma (HHV-8)				Yes
7	35	Female	Infected	Pneumocystis pneumonia				
8	49	Male	Uninfected	Diabetic and hypertensive nephropathy				
9	35	Female	Infected	Disseminated toxoplasmosis				
**10**	**27**	**Female**	**Infected**	**Pneumonia (Acinetobacter baumannii)**	**Present**	**Lung**	**Pyogenic Pneumonia**	**No**
11	41	Male	Uninfected	Meningitis (Streptococcus pneumoniae)				Yes
12	33	Male	Infected	CMV meningoencephalitis				
13	76	Female	Uninfected	Cerebral hemorrhage				
**14**	**17**	**Female**	**Infected**	**Meningitis (Mycobacterium tuberculosis)**	**Present**	**Lung, Brain**§	**Pyogenic meningoencephalitis**	**No**
15	43	Male	Infected	Pneumocystis pneumonia				
16	48	Male	Uninfected	Hepatocellular carcinoma				
**17**	**53**	**Female**	**Infected**	**Large B cell lymphoma**	**Present**	**Lung**§	**Pulmonary Necrotizing granulomas**	**No**
18	36	Female	Infected	Carcinoma (uterine cervix)				
19	62	Male	Uninfected	Cerebral infarction				
20	35	Male	Infected	Necrotizing adenoviral and cryptococcal pneumonia				Yes
**21**	**29**	**Female**	**Infected**	**Milliary tuberculosis**	**Present**	**Lung**§**, Brain, Liver**	**Disseminated necrotizing granulomas**	**No**
**22**	**61**	**Female**	**Uninfected**	**Tuberculous meningitis**	**Present**	**Lung, Brain**	**Pyogenic meningoencephalitis**	
23	30	Female	Infected	Cryptococcal meningitis				
24	74	Male	Uninfected	Gastric ulcer with upper gastrointestinal hemorrhage				
25	57	Female	Infected	Carcinoma (uterine cervix)				
**26**	**45**	**Female**	**Infected**	**Milliary tuberculosis**	**Present**	**Lung**§**, Brain, Liver**	**Disseminated necrotizing granulomas**	**No**
27	27	Female	Infected	Puerperal sepsis				
28	29	Female	Infected	Cryptococcal sepsis				
**29**	**32**	**Male**	**Infected**	**S.disgalactiae sepsis**	**Present**	**Lung**	**Pumonary necrotizing granulomas**	**Yes**
30	30	Female	Infected	Cerebral toxoplasmosis				

*By in-house RT PCR or LAMP.

^§^Positive Ziehl-Neelsen stain on histological slides.

**Table 2:** Xpert and conventional in house PCR results (including cycle threshold) for detection of DNA of *M.tuberculosis* in 8 cases presenting tuberculosis disease at death.

Case #	Lung tissue		Brain tissue		Liver Tissue	
	MTBCdetected withXpert (CT)	MTBCdetected within house-PCR	MTBCdetected withXpert (CT)	MTBCdetected within house-PCR	MTBCdetected withXpert (CT)	MTBCdetected within house-PCR
1	Yes (21.6)	Yes	No	No	No	No
10	no	Yes				
14	Yes (38.6)	No*	yes (16.3)	Yes		
17	Yes (19.7)	Yes				
21	Yes(22.2)	Yes				
22	Yes(32.6)	Yes	Yes (27.3)	Yes		
26	Yes(14.4)	Yes	Yes (30.7)	Yes	Yes (18.7)	Yes
29	Yes(21.0)	Yes				

Abbreviations: MTBC: Mycobacterium tuberculosis complex; CT: Cycle Threshold; PCR: polymerase chain reaction.

*DNA was not detected with in house PCR, but positive with LAMP.
